# STOBE: A Long-COVID Syndromic Study using Real-World data in Brazil

**DOI:** 10.1093/eurpub/ckac131.189

**Published:** 2022-10-25

**Authors:** H Cavalini, Z Qin, Y Zhen, J Q Shi, A Shetty, V Neves, G Delanerolle, P Phiri

**Affiliations:** 1 Universidade de Pernambuco, Petrolina, Brazil; 2 Southern University of Science and Technology, Shenzhen, China; 3 Alan Turing Institute, London, UK; 4 University College London Hospitals NHS, London, UK; 5 University College London, London, UK; 6 Nuffield Department of Primary Health Care Sciences, University of Oxford, Oxford, UK; 7 Research and Innovation Department, Southern Health NHS Foundation Trust, Southampton, UK; 8 Primary Care, Population Sciences and Medical Education, University of Southampton, Southampton, UK

## Abstract

**Background:**

The COVID-19 pandemic has changed the way infectious diseases are perceived. Global healthcare systems have faced challenges since the start of the COVID-19 epidemic, particularly in developing countries. Some individuals with an acute COVID-19 diagnosis have developed symptoms persisting beyond 90 days. Long-Covid is the new term for this syndrome (LC). LC, on the other hand, is poorly known and appears to cause a wide range of symptoms, particularly among Brazilian patients. As a result, utilizing retrospective data from patients in Petrolina, Brazil’s largest city in the northeast, we conducted an exploratory epidemiology study.

**Methods:**

A retrospective, cohort study design was used with a real-world dataset. The primary aim was to evaluate the prevalence of LC within Petrolina. The sample size was 1,164 LC patients. A comparative and subgroup analysis was conducted to evaluate demographics, comorbidities, clinical symptoms, and mortality. A k means model was used to assess disease severity using a clustering analysis based on the presence of comorbidities.

**Results:**

The prevalence of physical symptoms identified was 69·5%. The strongest physical symptom was fever with resultant of 64·09% followed by pain, 43·64%. The prevalence of autonomic and neurological symptomatology was 8·59% and 8·16% respectively. A higher prevalence of autonomic symptoms were reported among older men of Black and Caucasian in comparison to Pardo. Disease severity within the sample could be associated with the presence of comorbidities which were identified based on medication history. Pregnant women have high rate of comorbidities. 529 patients have at least one comorbidity and 28·73% of them are pregnant.

**Conclusions:**

It is useful to evaluate symptoms although a definitive diagnosis of LC is essential. This study provides insightful information around LC within a Brazilian population to develop better infection control protocols, as well as future management of similar pandemics.

**Key messages:**

• This study could potentially improve the prognosis and mortality among LC patients with comorbidities.

• Our findings could be combined with other regional datasets to predict pattern inferences of LC spread, prognosis and morbidity, including for multimorbidity and pregnant patients.

